# Angiomotin mutation causes glomerulopathy and renal cysts by upregulating hepatocyte nuclear factor transcriptional activity

**DOI:** 10.1002/ctm2.904

**Published:** 2022-06-13

**Authors:** Yaochun Zhang, Liangjian Lu, Zhenhua Hu, Yu Dai, Nurul Jannah Binti Ahmad, Jun Li Ng, Chang Yien Chan, Md. Zakir Hossain, Alwin Hwai Liang Loh, Jerrold M. Ward, Puay Hoon Tan, Sonia Davila, Vikrant Kumar, Walter Hunziker, Haishu Lin, Hui Kim Yap, Kar Hui Ng

**Affiliations:** ^1^ Department of Paediatrics Yong Loo Lin School of Medicine National University of Singapore Singapore; ^2^ Khoo Teck Puat‐National University Children's Medical Institute National University Health System Singapore; ^3^ Institute of Molecular and Cell Biology, Agency for Science Technology and Research (A*STAR) Singapore; ^4^ Department of Pharmacy National University of Singapore Singapore; ^5^ Cancer Science Institute of Singapore National University of Singapore Singapore; ^6^ Department of Anatomical Pathology Singapore General Hospital Singapore; ^7^ Global VetPathology Montgomery Village Maryland USA; ^8^ SingHealth Duke‐NUS Institute of Precision Medicine Singapore; ^9^ Cardiovascular and Metabolic Disorders Duke‐NUS Medical School Singapore; ^10^ SingHealth Duke‐NUS Genomic Medicine Centre Singapore; ^11^ Cancer and Stem Cell Biology Duke‐NUS Medical School Singapore; ^12^ Department of Physiology Yong Loo Lin School of Medicine National University of Singapore Singapore

Dear editor,

We identified angiomotin (*AMOT*) as a novel candidate gene for X‐linked recessive nephropathy associated with glomerular disease, tubulopathy and progressive kidney cystic disease. Transgenic rats carrying this mutation recapitulated the human phenotype to some extent. Orthogonal methods implicate the hepatocyte nuclear factor (Hnf) family of transcription factors, particularly Hnf4α and Hnf1β, in the development of *Amot* mutation‐induced nephropathy.

Monogenic causes involving more than 50 genes have been identified in 25%–30% of young patients with steroid‐resistant nephrotic syndrome (SRNS).^1^, ^2^ We report a family with X‐linked recessive early‐onset SRNS and Fanconi syndrome (Figure 1A–D). Exome sequencing identified NM_001113490.1:c.148A>G (p. S50G) variant in exon 1 of *AMOT*, which encodes *AMOT*. This variant causes the substitution of serine to glycine at the 50th position of AMOT‐P130 (Figures 1E,F and S1).

**FIGURE 1:**
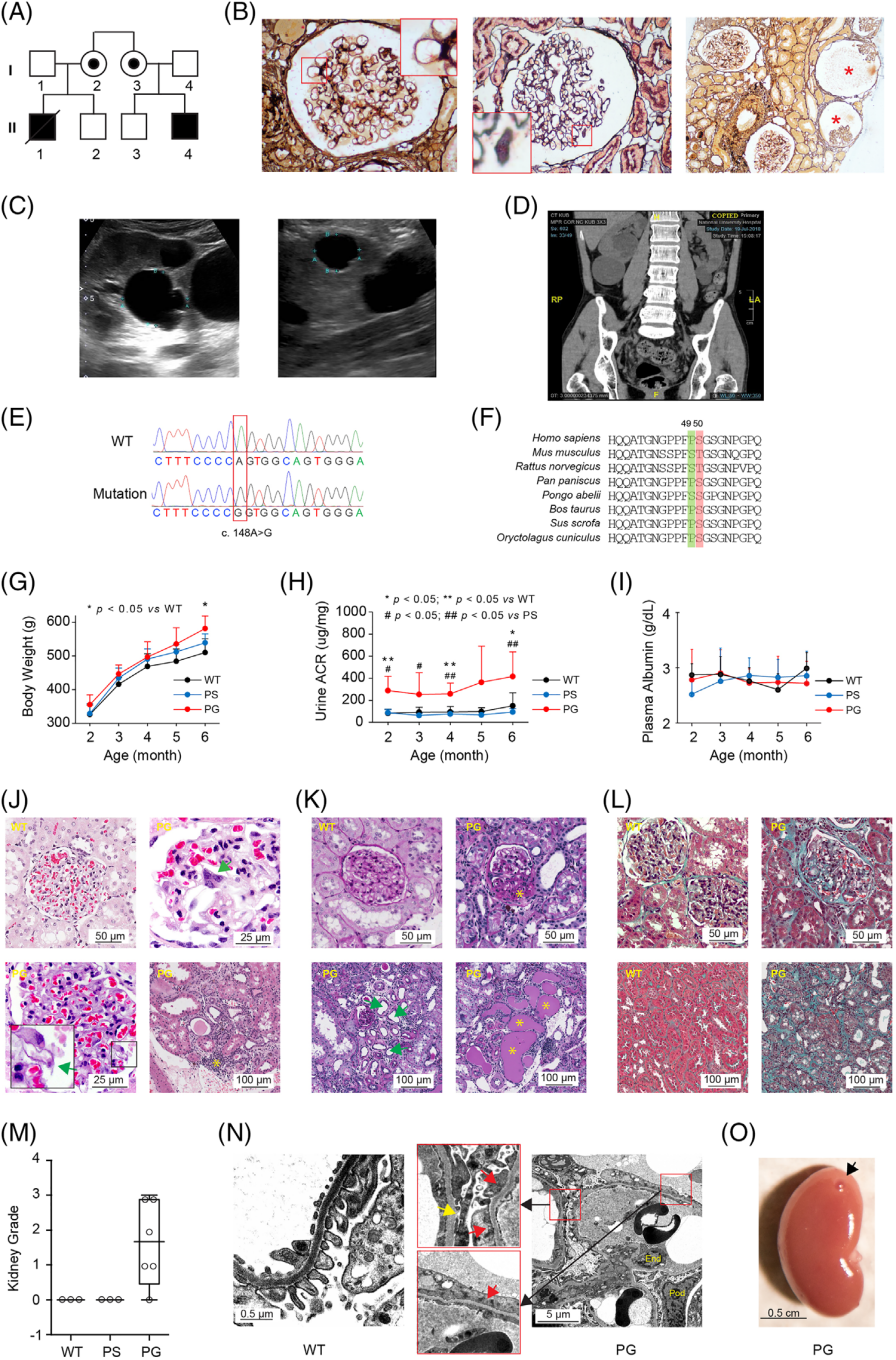
Angiomotin (*AMOT*) mutation caused nephropathy involving glomeruli and tubules in patients and transgenic rats. (A) A Chinese family with recessive early‐onset steroid‐resistant nephrotic syndrome (SRNS) associated with Fanconi syndrome. Patient II‐1 presented at 1.5 years old with SRNS due to membranous nephropathy and subsequently developed Fanconi syndrome. Patient II‐4 presented at age 11 months with SRNS and microscopic hematuria but no tubular dysfunction. (B) Renal biopsy from patient II‐4 showed glomerular basement membrane spikes (left, insert), indicating the development of membranous nephropathy. Glomerular cysts were present by age 15, evidenced by the basement membrane vacuoles (middle, insert) as well as glomerulocysts (right; red asterisks). Kidney cysts were first noticed on ultrasonography at 15 years old as a single kidney cyst measuring 1.1 cm. The number and size of the cysts rapidly progressed bilaterally. Large cysts up to 4.0 cm in size are shown by (C) ultrasonography (28 years old) and (D) computed tomography scan (32 years old; white arrows). (E) The *AMOT* missense genetic variant NM_001113490.1:c.148A>G found in whole‐exome sequencing of Patient II‐4 was confirmed via Sanger sequencing (boxed). (F) AMOT sequence alignment across different species. Amino acids at position 49 are highlighted in green, while amino acids at position 50 are highlighted in red. Although the altered amino acid at position 50 is not conserved, all species carry the hydroxyl amino acids serine (S) or threonine (T) at this site. At position 49, while *Rattus norvegicus, Mus musculus* and *Pongo abelii* carry a hydroxyl amino acid serine (S), *Homo sapiens* and other species carry a neutral amino acid proline (P). (G) PG rats had significantly higher body weights than WT rats at 6 months of age and (H) significantly higher urine albumin:creatinine ratios, compared to WT and PS rats from 2 months of age. (I) Serum albumin levels were, however, not different in PG, compared to WT and PS rats. (J) Representative light microscopy histology of glomeruli and tubulointerstitium of 6‐month‐old rats with hematoxylin and eosin staining. A representative image from a WT rat is shown on the top left. The PG rats developed various histological changes in glomeruli, including cellular hypertrophy (top right, green arrow), thickened glomerular basement membrane (bottom left, green arrow), and inflammatory infiltrates in the tubulointerstitium (bottom right, yellow asterisk). (K) With Periodic acid Schiff staining, PG rats showed focal segmental glomerulosclerosis characterised by increased mesangial matrix and obliteration of capillary lumina, as well as adhesion of glomerular tuft to Bowman's capsule (top right, yellow asterisk). Tubulointerstitial damage consisted mainly of tubular dilation (bottom left, green arrow) and cast deposits (bottom right, yellow asterisk). These features were not present in WT rats (top left). (L) Masson's trichrome staining revealed glomerular (top right) and tubulointerstitial fibrosis (bottom right) in PG rats, compared to physiological deposits of collagen in WT rats (top and bottom left). (M) Systematic scoring of kidney light microscopy changes was performed for 6‐month‐old WT, PS and PG rats. An overall histology score of 0 to 4 (0, normal; 1, minimal; 2, mild; 3, moderate; 4, severe abnormalities) was given for each rat. No histological kidney abnormalities were noted in WT and PS rats, while the PG rats had minimal to moderate histological changes (*n* = 3 for WT and PS rats, *n* = 6 for PG rats). (N) Electron microscopic kidney examination of 3‐month‐old WT rats revealed normal podocyte morphology with interdigitating patterns of the foot processes (left). In contrast, PG rats (right) had podocytes that showed extensive foot process effacement (red arrow) and detachment revealing areas with nude glomerular basement membranes (yellow arrow). Representative nuclei for podocyte (Pod) and glomerular endothelial cells (End) were labelled. (O) Macroscopic cyst seen on the kidney surface of a PG rat (black arrow) at 21 days old (scale bar: 0.5 cm).

To study the effects of this mutation in rats, we used the CRISPR/Cas9 system to substitute both serine and threonine residues at positions 49–50 with proline and glycine (termed ‘PG’ rat) or proline and serine (termed ‘PS’ rat), respectively (Figure S2). PG rats developed higher body weights and albuminuria (Figure 1G,H). At 6 months old, PG rats developed focal segmental glomerulosclerosis, tubular dilatation, tubulointerstitial inflammation and fibrosis (Figure 1J–L). Systematic scoring of the light microscopic changes revealed no kidney abnormalities in wild type (WT) and PS rats, while the PG rats had minimal to moderate changes (Figure 1M). We then examined the ultrastructural changes at 3 months old. While WT rats had normal morphology, the podocytes in PG rats showed extensive foot process effacement and detachment, revealing areas with nude glomerular basement membranes (Figure 1N). At 21 days old, macroscopic cysts were also noted on the kidney surfaces in PG rats (8/11 = 72.7%; Figure 1O).

In *ex vivo* podocytes, the PG mutation disrupted the expression of F‐actin, causing abnormal formation of stress fibers (Figures 2A and S3), and reduced cell stiffness (Figure S4). Abnormal zonula occludens‐1 (Zo‐1) expression and distribution were observed in mutant rat kidneys (Figure 2B), *ex vivo* podocytes (Figure 2C) and proximal convoluted tubule cells (PCT; Figure 2D). In addition, the expression of occludin, another tight junction protein, was reduced in *Amot* mutant PCT cells (Figure 2E). In fluorescein isothiocyanate (FITC)‐albumin flux analysis, FITC‐albumin flux across the PCT monolayer was increased (Figure 2F,G) in the PG mutant cells, implying that the PG mutation resulted in perturbation in the tight junctions.

**FIGURE 2:**
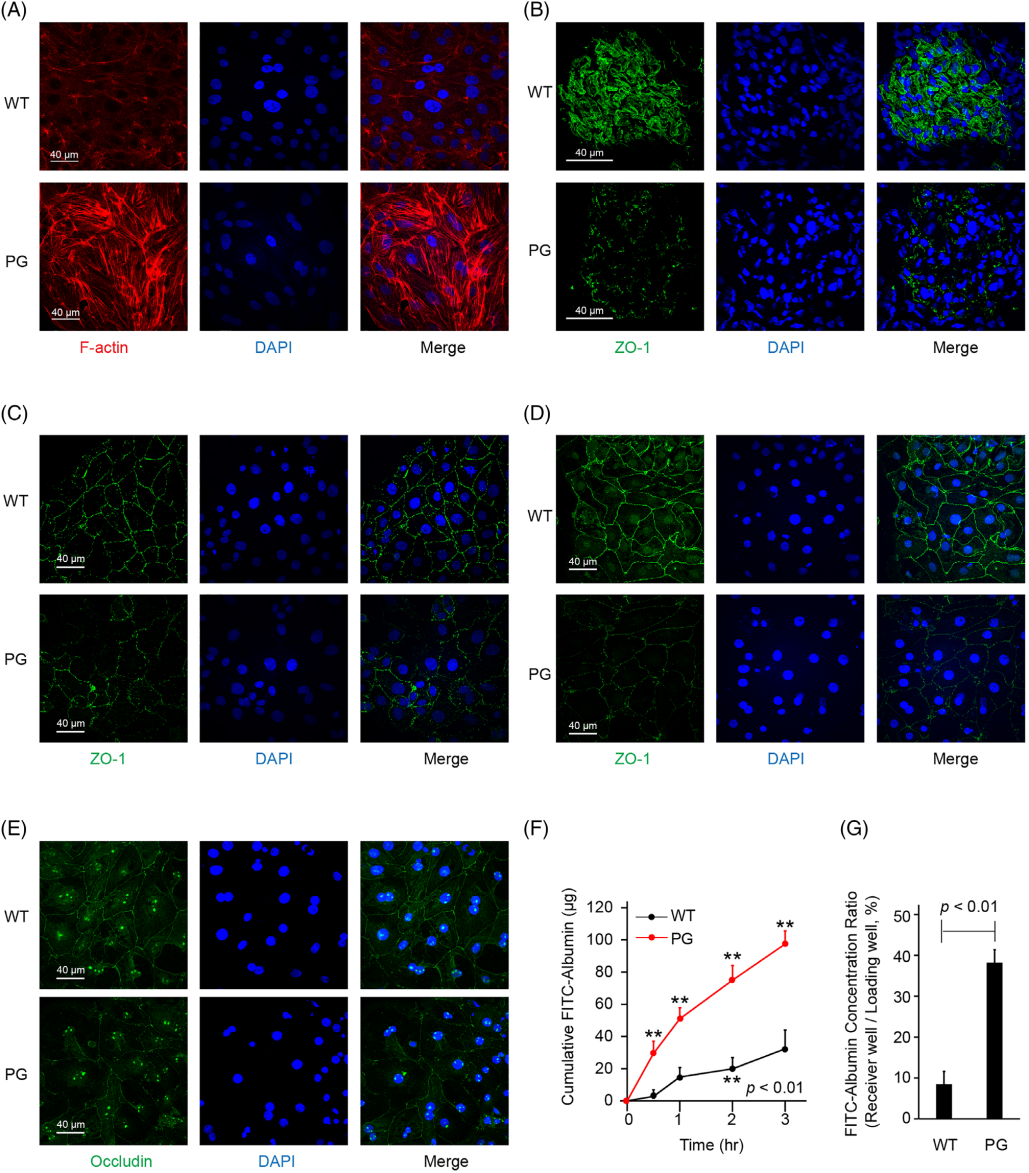
*Amot* PG genetic alteration increased stress fiber formation and perturbed tight junction. (A) Immunofluorescence of *ex vivo* podocytes by phalloidin staining. The PG genetic alteration increased and disrupted the expression of F‐actin, causing abnormal stress fiber formation. (B) The expression and distribution of the tight junction‐related protein Zo‐1 were abnormal in the mutant PG rat kidneys, *ex vivo* (C) podocytes and (D) proximal convoluted tubular cells. (E) The expression of occludin, another tight junction protein, was reduced in *Amot* mutant proximal convoluted tubule (PCT) cells. (F) Fluorescein isothiocyanate (FITC)‐albumin flux across the PCT monolayers was evaluated. FITC‐albumin loaded on the loading well was allowed to diffuse across the cell monolayer to the receiver well. The cumulative FITC‐albumin concentration in the receiver well significantly increased in the PG group, compared to the WT over time, suggesting increased permeability in the PG PCT monolayer. Each dot represents the average of four independent experiments. (G) FITC‐albumin concentration ratio between the two sides of the PCT monolayer after 4 h, calculated as [albumin]_receiver well_ / [albumin]_loading well_, was compared between PG and WT monolayers. The increased FITC‐albumin ratio in the PG monolayer implied increased permeability and perturbation in the tight junctions.

AMOT interacts with multiple proteins of the Hippo signalling pathway, including the transcriptional effector Yes‐associated protein.^3^ Our studies, however, suggested that the Hippo signalling pathway is not the crucial mechanism downstream of the *Amot* mutation (Figure S5).

Single‐cell RNA sequencing (RNA‐Seq) revealed relatively high transcriptional levels of AMOT in podocyte and proximal tubule segment S1 cells. Thus, to decipher disease pathogenesis, RNA‐Seq was performed on freshly isolated PCT cells since they are more easily isolated than podocytes. Hypergeometric Optimisation of Motif Enrichment (HOMER) was then applied to detect the transcription factor binding motifs in the promoters of the up‐ and down‐regulated genes (Figure 3A–D). Here, we showed that Hnfs, namely Hnf1, Hnf1β and Hnf4α, were enriched in the promoters of PG‐upregulated genes, suggesting that the *Amot* mutation caused an upregulation of Hnfs. Of note, HNF1A and HNF1B are associated with Fanconi syndrome and kidney cystic diseases in humans.^4^, ^5^ Gene set enrichment analysis (GSEA) of Hnf4α target genes (Table S3) corroborated the motif enrichment analysis, as it suggested that Hnf4α target genes were upregulated in PG rats (Figure 3E). To confirm this finding, the differential expression of four upregulated genes (*Acox2, Aldh2, Aldob* and *Otc*) known to be Hnf4α target genes was validated by quantitative polymerase chain reaction (Figure 3F).

**FIGURE 3:**
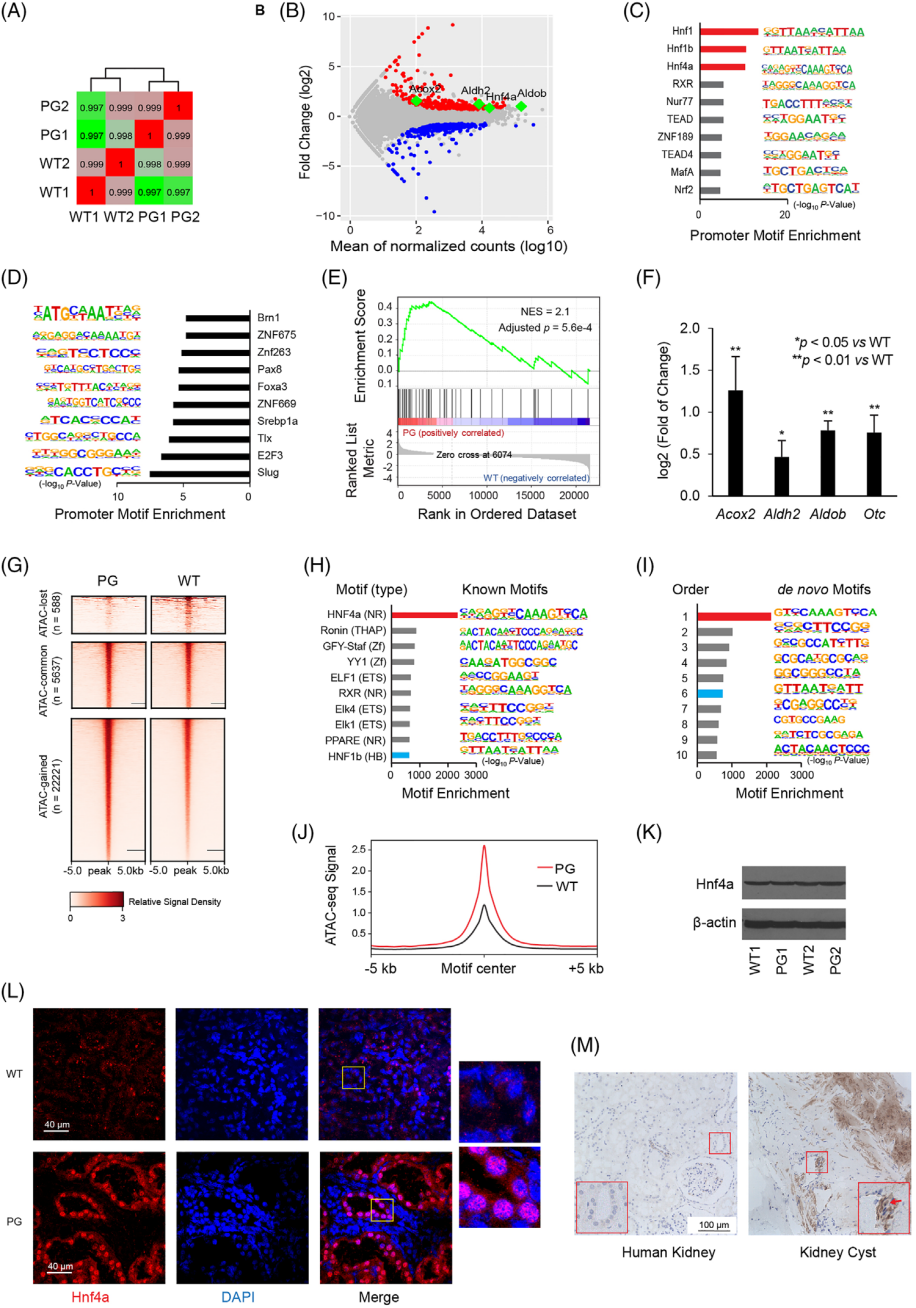
Orthogonal methods implicated Hnf4α in the development of *Amot* mutation‐induced nephropathy in rats. (A) Heatmap of Pearson correlation between the RNA sequencing (RNA‐Seq) samples. Hierarchical clustering was used to group similar samples into two clusters. Correlation coefficients between each two samples are shown in the boxes crossed by the two samples. Correlation coefficient values are reflected by the box colours (the lowest to the highest in value, green to red in colour). (B) MA plot of mRNA reads on proximal convoluted tubular cells from WT and PG rats. A total of 231 genes were induced (red dots), and 202 genes were suppressed (blue dots) in PG rats (false discovery rate (FDR) ≤ 0.05, fold change (FC) ≥ 2). Four representative differentiated genes that were known to be hepatocyte nuclear factor 4α (Hnf4α) targets are labelled in green. (C) Motif enrichment by Hypergeometric Optimisation of Motif EnRichment (HOMER) revealed that the HNF family transcription factors, namely Hnf1, Hnf1β and Hnf4α (red bars) were enriched in the promoter regions of upregulated genes in PG rats (D) but not in the promoter regions of downregulated genes in PG rats. (E) Gene set enrichment analysis showed a normalized enrichment score (NES) of 2.1 between PG and WT rats (adjusted *p* = 0.0006), implying that the expression of Hnf4α target genes is higher in PG rats. (F) qPCR of four representative Hnf4a target genes, *Acox2*, *Aldh2*, *Aldob* and *Otc*. Glyceraldehyde‐3‐phosphate dehydrogenase (GADPH) levels were used to normalize for the amounts of cDNA loaded. Relative gene expressions were calculated using the comparative Ct method. (G) Landscape of chromatin accessibility in PCTs isolated from WT and PG rat kidneys. A total of 22 221 regions gained assay for transposase‐accessible chromatin (ATAC) signals (ATAC‐gained), and 588 regions lost ATAC signals (ATAC‐lost) in PG, while 5637 regions maintained similar ATAC accessibility between WT and PG (ATAC‐common). (H, I) Enrichment of both known and *de novo* transcription factor motifs in the ATAC‐gained regions was performed. The nuclear transcription factor Hnf4α was the most enriched transcription factor for both known and *de novo* motifs (red bars). Another Hnf family member, Hnf1β, was also among the top 10 enriched motifs (blue bars). (J) The average ATAC‐seq signal around the Hnf4α motif site was increased in PG, compared to WT rats, implying increased chromatin accessibility across the whole genome in PG rats. (K) Western blot analysis of protein samples isolated from rat PCTs showing that Hnf4α protein levels were not upregulated in PG rats. (L) Immunofluorescence of Hnf4α in rat kidneys revealed increased nuclear recruitment of Hnf4α in the PG rat tubules compared to WT. The PG rat tubules were dilated compared to WT, thus accounting for the discrepant diameters of the tubules between the WT and PG rats. (M) Immunohistochemistry of HNF4α in the patient's kidney cyst tissue showed extensively increased HNF4α expression, as well as increased nuclear recruitment, compared to normal human kidney.

The genome‐wide chromatin accessibility landscape was profiled by Assay for Transposase‐Accessible Chromatin using sequencing (ATAC‐Seq) in freshly isolated PCT cells (Figures 3G and S6). Putative transcription factor motifs in the ATAC‐gained regions were identified with HOMER. Again, the nuclear transcription factor Hnf4α was enriched for both known motifs and *de novo* motifs. Another Hnf family member, Hnf1β, was also among the top 10 enriched motifs (Figure 3H,I). On further analysis of ATAC‐seq data around Hnf4α motif sites, we confirmed its increased chromatin accessibility across the whole genome in the PG rat (Figure 3J). HNF4α is the master regulator of many hepatocyte‐specific genes.^6^ Neither the WT nor the mutant AMOT bound directly to the HNF4α protein (Figure S7). Activation of the Hnf4α pathway in PG rat kidneys occurred in the absence of upregulated Hnf4α protein levels (Figure 3K). Instead, the *AMOT* mutation resulted in nuclear recruitment of HNF4α in PG rat tubules, corroborating findings in the patient's (II‐4) kidney cyst tissue (Figure 3L,M). The importance of HNF4α activation to the PG phenotype is further supported by stress fiber formation and altered ZO‐1 distribution when kidney cell lines are treated with Benfluorex, a known HNF4α activator (Figure S8).^7^

Considering that the GSEA of the differentially expressed genes revealed metabolic pathway alterations, we performed metabolic profiling of the rat plasma as an exploratory analysis to identify the metabolic pathways involved (Figure 4A). A total of 37 metabolites were identified as critical metabolites as a result of the *Amot* mutation (Table. S4). Pathway enrichment of the critical metabolites with MetaboAnalyst showed that the pentose and glucuronate interconversion pathway and the tricarboxylic acid (TCA) cycle were the most impactful metabolic pathways (Figures 4B and S9). The main altered molecules that account for these metabolic pathways are shown in Figure 4C,D. Both of these metabolic pathways were also significantly altered in the KEGG pathway analysis of the differentially expressed genes identified in RNA‐Seq (Figure 4E).

**FIGURE 4:**
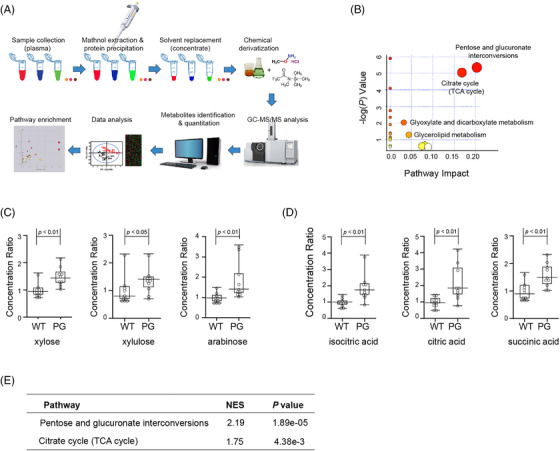
Metabolic profiling of rat plasma revealed that *Amot* PG genetic alteration caused changes in several metabolic pathways. (A) Flow chart showing the steps involved in the metabolic profiling of the rat plasma on a gas chromatography mass spectromery/mass spectrometry (GC‐MS/MS) platform. (B) Metabolic pathway enrichment using MetaboAnalyst. Pentose and glucuronate interconversions and the TCA cycle were enriched as the most impactful metabolic pathways associated with Amot PG alteration. The node colour was based on its *p‐*value, and the node radius was determined based on their pathway impact values. (C) Rat plasma concentrations of xylose, xylulose and arabinose, which are the key altered molecules in pentose and glucuronate interconversions, and (D) isocitric acid, citric acid and succinic acid, which are the key altered molecules in the TCA cycle. The plasma levels of all these metabolites were significantly higher in the PG rats compared to the WT rats (median ± interquartile range (IQR), *n* = 10 for each group). (E) Kyoto Encyclopedia of Genes and Genomes (KEGG) pathway enrichment of the pentose and glucuronate interconversions pathway and TCA cycles with the differentially expressed genes identified in RNA‐Seq.

Rodent models of several genes associated with glomerulotubular nephropathy (PAX2, CRB2 and FAT1) have revealed different pathophysiological mechanisms.^8^, ^9^, ^10^ By implicating HNFs and downstream metabolic changes in AMOT nephropathy, we add to the current pathogenetic understanding of glomerulotubular nephropathies.

In conclusion, we report a putative novel role of *AMOT* in causing glomerulotubular nephropathy in patients and rats, possibly by regulating the HNF4α pathway and the subsequent metabolic pathways.

## CONFLICT OF INTEREST

The authors declare no conflicts of interest.

## Supporting information

Additional supporting information may be found in the online version of the article at the publisher's website.

## References

[1] Preston R , Stuart HM , Lennon R . Genetic testing in steroid‐resistant nephrotic syndrome: why, who, when and how? Pediatr Nephrol. 2019;34(2):195‐210. 29181713 10.1007/s00467-017-3838-6 PMC6311200

[2] Sadowski CE , Lovric S , Ashraf S , et al. A single‐gene cause in 29.5% of cases of steroid‐resistant nephrotic syndrome. J Am Soc Nephrol. 2015;26(6):1279‐1289. 25349199 10.1681/ASN.2014050489 PMC4446877

[3] Paramasivam M , Sarkeshik A , Yates JR, 3rd , Fernandes MJ , McCollum D . Angiomotin family proteins are novel activators of the LATS2 kinase tumor suppressor. Mol Biol Cell. 2011;22(19):3725‐3733. 21832154 10.1091/mbc.E11-04-0300 PMC3183025

[4] Improda N , Shah P , Guemes M , et al. Hepatocyte nuclear factor‐4 alfa mutation associated with hyperinsulinaemic hypoglycaemia and atypical renal fanconi syndrome: expanding the clinical phenotype. Horm Res Paediatr. 2016;86(5):337‐341. 27245055 10.1159/000446396

[5] Bingham C , Bulman MP , Ellard S , et al. Mutations in the hepatocyte nuclear factor‐1beta gene are associated with familial hypoplastic glomerulocystic kidney disease. Am J Hum Genet. 2001;68(1):219‐224. 11085914 10.1086/316945 PMC1234916

[6] Ko HL , Zhuo Z , Ren EC . HNF4alpha combinatorial isoform heterodimers activate distinct gene targets that differ from their corresponding homodimers. Cell Rep. 2019;26(10):2549‐2557. 30840880 10.1016/j.celrep.2019.02.033

[7] Lee SH , Athavankar S , Cohen T , et al. Identification of alverine and benfluorex as HNF4alpha activators. ACS Chem Biol. 2013;8(8):1730‐1736. 23675775 10.1021/cb4000986 PMC3922238

[8] Dressler GR , Woolf AS . Pax2 in development and renal disease. Int J Dev Biol. 1999;43(5):463‐468. 10535325

[9] Slavotinek A , Kaylor J , Pierce H , et al. CRB2 mutations produce a phenotype resembling congenital nephrosis, Finnish type, with cerebral ventriculomegaly and raised alpha‐fetoprotein. Am J Hum Genet. 2015;96(1):162‐169. 25557780 10.1016/j.ajhg.2014.11.013 PMC4289687

[10] Gee HY , Sadowski CE , Aggarwal PK , et al. FAT1 mutations cause a glomerulotubular nephropathy. Nat Commun. 2016;7:10822. 26905694 10.1038/ncomms10822 PMC4770090

