# Supplementary material for: Angiomotin mutation causes glomerulopathy and renal cysts by upregulating hepatocyte nuclear factor transcriptional activity
Source: Clin Transl Med. 2022 Jun 13;12(6):e904. doi: 10.1002/ctm2.904 (PMC9191868; doi:10.1002/ctm2.904)
Supplement: Supplementary file 1 — Additional supporting information may be found in the online version of the article at the publisher's website. [file CTM2-12-e904-s001.docx]

**Supplementary Material**

|  | | Pg No |
| --- | --- | --- |
| **Method** | | 2 |
| **Tables** | |  |
|  | Table S1. Antibodies used in this study. | 14 |
|  | Table S2. Primer sequences. | 15 |
|  | Table S3. List of Hnf4α target genes in rat. | 16 |
|  | Table S3. Critical metabolites in rat plasma associated with *Amot* mutation. | 17 |
| **Figures** | |  |
|  | Figure S1. Structure of angiomotin proteins and expression of AMOT-P130 in kidneys. | 18 |
|  | Figure S2. Establishment of *Amot* mutant rat lines. | 19 |
|  | Figure S3. Immunofluorescence of *ex vivo* podocytes by phalloidin staining. | 20 |
|  | Figure S4. Amot PG genetic alteration reduced cell stiffness on *ex vivo* podocytes. | 21 |
|  | Figure S5. Hippo signaling pathway was not affected by the *AMOT* mutation. | 22 |
|  | Figure S6. ATAC-seq on freshly isolated proximal convoluted tubular cells from rats. | 23 |
|  | Figure S7. Co-immunoprecipitation of AMOT-130 with HNF4α. | 24 |
|  | Figure S8. Hnf4α activation caused abnormal stress fiber formation and tight junction disturbance in wild-type kidney cell lines. | 25 |
|  | Figure S9. Principal component and partial least squares discriminant analysis of metabolic profile in rat plasma. | 26 |
| **Reference** | | 27 |

**Method**

## ***Experimental animals***

Sprague-Dawley (SD) rats, restricted flora grade, were purchased from *InVivos* (Singapore). They were provided with a standard diet and water *ad libitum*. The room was kept on a 12/12 h light/dark cycle at a temperature of 23 ± 1 °C and relative humidity of 50 ± 10%.

***Rationale for using PG and PS genetic alterations in the rats***

The Amot mutation observed in the affected humans was p.S50G, representing a serine to glycine substitution. As demonstrated in Figure 1F, both the 49th and 50th residue differed in rodents compared to humans, i.e. ST (rat) rather than PS (humans).

In order to avoid possible potential functional compensation by the hydroxyl serine at position 49 and to better represent the human genetic variant, we substituted both hydroxyl residues serine (S) and threonine (T) at positions 49-50 with two hydrophobic counterparts proline (P) and glycine (G) respectively (termed “PG” rat). To ensure the phenotypes observed in PG rats are solely due to the substituted glycine at position 50 and to better represent the human wild-type counterpart, we created a control rat model with residues at positions 49-50 substituted as proline (P) and serine (S) (termed “PS” rat).

In summary, to model only the effect of an p.S50G mutation, we “humanised” the rat Amot protein by substituting ST with PS, and then compared this “humanised” PS rat to the mutant PG rat. This strategy increased the confidence that the effects of the human p.S50G mutation in rats are studied. Any functional changes we observe in PG rats compared to PS rats will be solely due to the G substitution at position 50.

***Microinjection of CRISPR/Cas9 system***

Using CRISPR/Cas9 system, we edited the AMOT gene in rat. The microinjection of fertilized rat eggs was described previously.^1^ In brief, prepubescent female rats (4-5 weeks old) were injected with 30 IU pregnant mare serum gonadotropin (PMSG; Sigma-Aldrich), followed by an injection of 20 IU of human chorionic gonadotropin (hCG; Sigma-Aldrich) 48 h later, and immediately mated with SD males. Fertilized one-cell stage embryos were collected from oviducts on the next day and cultured in KSOM (Millipore) at 37°C, 5% CO_2_ for 2 h and then prepared for microinjection. Microinjections were performed using a Nikon Microinjection System under standard conditions. To create *Amot* mutant rats, a mixture of Cas9 endonuclease (10 ng/µl; Toolgen, Korean), sgRNA (8 ng/µl) and a single-stranded donor oligonucleotide (3 ng/µl), which encodes the PG or PS genetic alteration of angiomotin, were injected into the rat zygotes. Surviving embryos were implanted in the oviduct of pseudo-pregnant females (0.5 dpc) on the same day and allowed to develop to full term.

## ***Genotype and phenotype analysis***

Genomic DNA was extracted from rat tail biopsy samples using E.Z.N.A.^®^ Tissue DNA Kit (Omega) as per manufacture’s protocol. A 500-bp oligonucleotide that covered the mutation site was amplified and the polymerase chain reaction (PCR) products were digested with AvaI for restriction fragment length polymorphism (RFLP) analysis or subjected to Sanger sequencing. Animal body weights were recorded, serum was collected *via* tail vein, and 24-hour urine was collected using metabolic cages at one-month intervals. Urine albumin levels were measured using a direct sandwich ELISA system (anti-rat albumin serum:rat serum:horseradish peroxidase (HRP) conjugated anti-rat albumin antibody). Urine creatinine levels were determined using the kinetic Jaffe method according to manufacturer’s instructions (ELITech, USA).

## ***Kidney histology***

Rat kidneys were fixed with 4% paraformaldehyde, dehydrated in alcohol gradient, embedded in paraffin and then serially sectioned into 3 µm thickness sections. After deparaffinization and rehydration, hematoxylin and eosin, periodic acid Schiff and Masson's trichrome staining were performed. The kidney histology (light microscopy) were graded by a blinded veterinary pathologist. For each rat, the histology was evaluated for abnormalities in glomeruli including podocytes, glomerular basement membranes and tubulo-interstitium. An overall histology score of 0 to 4 (0, normal; 1, minimal; 2, mild; 3, moderate; 4, severe abnormalities) was given for each rat. Immunohistochemistry was performed on BondMax® (Leica Biosystems). Microscopic examinations were carried out using a Leica DM RX® light microscope. For transmission electron microscopy, kidney cortex samples were primarily fixed with 2.5% glutaraldehyde, and then followed by 1% OsO4 post-fixation. After dehydration in ethanol and acetone, kidney tissues were embedded in Epon resin. Ultrathin sections were incubated with lead citrate in uranyl acetate and then visualized with a JEOL JEM-1010® electron microscope.

## ***Rat glomeruli isolation and culture***

Rats were anesthetized by an intraperitoneal injection of sodium pentobarbital (50 mg/kg body weight). A cut was made in the vena cava and the kidneys were perfused with 12 ml of phosphate-buffered saline containing 2.4×10^7^ Dynabeads® (Φ4.5 µm; ThermoScientific) and 12 mg of iron powder (Φ6 µm; Sigma-Aldrich) *via* abdominal aorta. The kidney cortices were dissected and cut into small pieces (1-2 mm cubes) with a surgical blade in Hanks’ balanced salt solution (HBSS). The tissues were digested in collagenase solution containing 1 mg/ml collagenase A and 0.2 mg/ml deoxyribonuclease I in HBSS at 37 ^0^C for 30 min with gentle agitation. The collagenase-digested tissues were gently pressed through a 100 µm cell strainer (Sigma-Aldrich) using a flattened pestle. Glomeruli containing Dynabeads® were gathered by a magnetic particle concentrator and washed with phosphate-buffered saline. Finally, collected glomeruli were loaded onto a 70-µm cell strainer to wash away tubules, single cells, small debris and fragmented glomeruli.^2^ Intact glomeruli on the cell strainer are collected and cultured on type I collagen-coated culture dishes or glass coverslips in RPMI-1640 (ThermoScientific) containing 5% fetal bovine serum (ThermoScientific), supplemented with 0.5% insulin-transferrin-selenium-sodium pyruvate (ITS-A) liquid media supplement (ThermoScientific), 100 U/ml penicillin, and 100 mg/ml streptomycin. The isolated glomeruli were incubated in a 37 ^0^C humidified incubator with 5% CO_2_.

## ***Rat proximal convoluted tubular cell isolation and culture***

Primary rat proximal convoluted tubular cells (PCTs) were isolated using protocol as described previously.^3,4^ The kidney cortices were dissected and cut into small pieces (1-2 mm cubes) with a surgical blade in HBSS. The tissues were digested in 1 mg/ml collagenase IV (Sigma-Aldrich) at 37 ^0^C for 60 min with gentle agitation. The digested tissues were filtered with a 70 µm cell strainer (Sigma-Aldrich) and spun down. After removal of the supernatant, the pellets were re-suspended by 42% Percoll® (GE Healthcare Life Sciences) and subjected to gradient centrifugation at 31,000 *g*, 4 ^0^C for 30 min. The 4^th^ layer was collected, washed and cultured on type I collagen-coated culture dishes or glass coverslips in DMEM/F12 (ThermoScientific) containing 2% fetal bovine serum (ThermoScientific), supplemented with 0.5% insulin-transferrin-selenium-sodium pyruvate (ITS-A) liquid media supplement (ThermoScientific), 100 U/ml penicillin, and 100 mg/ml streptomycin. Cells were incubated in a 37 ^0^C humidified incubator with 5% CO_2_.

***Cell lines***

HEK293, HK-2 and MDCK were purchased from American Type Culture Collection (ATCC; USA). HEK293 and MDCK were cultured in Gibco Dulbecco's Modified Eagle Medium (DMEM; ThermoScientific) containing 10% fetal bovine serum (ThermoScientific), supplemented with 100 U/ml penicillin, and 100 mg/ml streptomycin. HK-2 cells were cultured in Keratinocyte Serum Free Medium (K-SFM; ThermoScientific) containing 0.05 mg/ml bovine pituitary extract (BPE) and 5 ng/ml human recombinant epidermal growth factor (EGF), supplemented with 100 U/ml penicillin, and 100 mg/ml streptomycin. The cells were incubated in a 37 ^0^C humidified incubator with 5% CO_2_. To activate HNF4α, HK-2 and MDCK cells were treated with 20 µM of Benfluorex hydrochloride (Sigma-Aldrich) for 24 hr before being fixed for immunofluorescence staining.

## ***Immunofluorescence staining***

Cells and tissue sections were mounted onto glass slides, fixed in 4 % formaldehyde and permeabilized with 0.3% Triton X-100. After blocking in 5% bovine serum albumin, samples were incubated with primary antibodies and fluorescent conjugated second antibodies successively. Nuclei were counter stained with DAPI (0.1 μg/ml in phosphate-buffered saline). Images were captured using FluoView FV1200® confocal laser scanning microscope (Olympus) and analyzed with FluoView Viewer® software.

***Albumin-FITC Flux Assay***

Albumin permeability across the monolayers formed by proximal convoluted tubular cells was measured using protocol as described previously.^5^ In brief, freshly isolated PCTs from WT or PG rats were seeded onto the insert membrane of 24-well Transwell® (Corning). After cells grew to 100% confluence (Day 4), 0.3 ml of DMEM/F12 medium (ThermoScientific) containing 0.5 mg/ml of FITC conjugated albumin (ThermoScientific) was loaded into the insert wells, while the receiver compartments were filled with 0.5 ml of DMEM/F12 medium (ThermoScientific) containing 0.5 mg/ml albumin. 100 μl of medium was collected from each receiver compartment at 0.5, 1, 2 and 3 hr for albumin flux assay. 100 μl of DMEM/F12 medium (ThermoScientific) containing 0.5 mg/ml albumin were added into the receiver compartments after each collection. Albumin-FITC were measured under the excitation wavelength of 490 nm and emission wavelength of 525 nm and concentrations were calculated from the standard curve.

***RNA-Seq and ATAC-Seq***

For RNA-Seq, total RNA was isolated from rat proximal convoluted tubules using the RNAqueous^®^ total RNA Isolation Kit (Thermo Fisher Scientific; AM1931) according to the manufacturer’s instructions. Ribosomal depletion, RNA-Seq libraries construction, as well as Illumina sequencing were performed by NovogeneAIT Genomics (Singapore). For ATAC-Seq, proximal convoluted tubules were isolated from rat kidneys and snapped frozen in liquid nitrogen for at least 1 h. Cell lysis, nuclei extraction, transposition reaction, library generation, as well as ATAC sequencing, were performed by NovogeneAIT Genomics (Singapore).

### *RNA-seq data analysis*

Paired-end raw sequencing reads were trimmed with Trim Galore (version 0.4.2_dev; <https://www.bioinformatics.babraham.ac.uk/projects/trim_galore/>) with the following parameters: -*trim-n*-*paired*. Cleaned reads were then mapped to the Rat Rn5 reference genome, guided by the gene model from the NCBI Rattus norvegicus Annotation Release 104 using the RSEM pipeline (version 1.1.11).^6^ The reference genome and gene model files were obtained from Illumina iGenome website (<http://igenomes.illumina.com.s3-website-us-east-1.amazonaws.com/Rattus_norvegicus/NCBI/Rnor_5.0/Rattus_norvegicus_NCBI_Rnor_5.0.tar.gz>). DeSeq2 (version 1.16.1)^7^ was applied to differential gene expression analyses with default settings. Genes were considered to be differentially expressed if they showed at least a twofold difference in expressions with an adjusted *P* value ≤ 0.05 after correcting for multiple testing by FDR (Benjamini and Hochberg FDR). MA plot was generated using ggplot2 in R.

### *Sample clustering*

Read counts were obtained for each sample using the RSEM pipeline described above. A log_2_ transformation was applied after adding one pseudo-count (that is, log_2_[count + 1]). A sample distance matrix was then calculated based on the correlation similarity method, using the Dist function from the amap package (<https://cran.r-project.org/web/packages/amap/index.html>), and the hclust function from the R standard statistical package was used for clustering by the hierarchical complete linkage clustering method.

*Gene set enrichment analysis*

Gene Set Enrichment Analysis (GSEA) against Kyoto Encyclopeida of Genes and Genomes (KEGG) ^8,9^ were performed using the function of gseKEGG in the package clusterProfiler.^10^ For GSEA analysis, *P* values were calculated based on one million permutations. Pathways were considered as significant if the FDR-corrected *P* value was < 0.05.

### *ATAC-seq data analysis*

#### *ATAC-Seq peak calling and annotation*

ATAC-Seq is a technique that identifies accessible DNA regions by sequencing hyperactive mutant Tn5 Transposase cleaved and tagged open regions of the genome. ^11^ Sequencing reads was used to infer regions of increased accessibility as well as to map regions of transcription factor binding sites. Paired-end raw sequencing reads were trimmed with Trim Galore to remove adaptors and trim low-quality reads. Cleaned reads were then mapped to the Rat Rn5 reference genome with Bowtie 2 (version 2.2.9) ^12^ with the following parameters: -*N 1–L 25–X 2000–no-mixed–no-discordant*. Reads mapped to mitochondria sequence were filtered out, and only uniquely mapped reads of mapping quality MAPQ >= 10 were kept. Mapped reads were further sorted and PCR duplicates were removed using SAMtools (version 1.4). ^13^ Reads were merged for replicates to generate the condition level BAM files and differential ATAC peaks were called between different genotypes (PG versus WT). This was performed using the callpeak function of MACS2 to generate bedgraph files (with the parameters -B-nomodel-shift -100-extsize 200 -keep-dup all), on which the bdgdiff subcommand was applied (with the parameters -l 500-g 250) to call ‘differential peaks’. ATAC-gained and ATAC-lost regions were annotated to nearest genes within 100 kb neighborhood.

#### *ATAC coverage bigwig file generation*

Replicate-merged bedgraph files were generated using the MACS2 callpeak function (with the parameters -*B-SPMR-nomodel-shift-100-extsize 200-t replicate1.bam replicate2.bam -keep-dup all*). These bedgraph files were subsequently converted to bigwig format using the bedGraphToBigWig function from Kent informatics. (http://hgdownload.soe.ucsc.edu /downloads.html#source_downloads). Metagene heatmap and profile plots were generated using the deepTools suite.^14^

### *Motif discovery and enrichment analysis*

Homer2 (Hypergeometric Optimization of Motif EnRichment, version 4.9.1; University of California San Diego) was used for motif discovery and enrichment analysis. For motifs across promoters, the search space was defined as a 4 kilobase (kb) window centred at the transcription start site (*findMotifs.pl geneInput.txt rat out/ -start -2000 -end 2000 -len 8,12 -p 10*). For motifs at PG ATAC-gain and ATAC-lost regions, the search space was defined as the whole region (*findMotifsGenome.pl regionInput.bed rn5 out/ -size given-p 10*).

## ***Real-time PCR***

Total RNA was isolated using the RNAqueous^®^ total RNA Isolation Kit (Thermo Fisher Scientific, USA) and reverse transcribed into cDNA using Maxima First Strand cDNA Synthesis Kit® (Thermo Fisher Scientific, USA) according to the manufacturer’s instructions. Gene expression was quantified by real-time PCR using LightCycler^®^ 480 Instrument II (Roche Diagnostics). The fluorescent melting curves were analysed using the LC480 Gene Scanning software v1.5® (Roche Diagnostics). The primer sequences used are given in Supplementary Material: Table S2. GADPH levels were used to normalize for the amounts of cDNA loaded. Relative gene expressions were calculated using the comparative Ct method.

***YAP/TAZ-responsive promoter driving luciferase reporter assay***

HEK293 cells that were seeded in 6-well culture plates and at approximately 60% confluency were cotransfected with 1.0 μg YAP/TAZ luciferase reporter plasmid (Addgene, #34615), 0.05 μg pRL-CMV plasmid (Promega, USA), and 1.0 μg pCruz-HA-AMOT plasmid (WT or p.S50G) per well for 24 h. Firefly and renilla luciferase activities were assayed using the Dual Luciferase® Assay System (Promega, USA) and results were expressed as firefly luciferase activity normalized to renilla luciferase activity.

***Co-immunoprecipitation (Co-IP) assay***

HEK293 cells that were seeded in 10 cm petri dish and at approximately 80% confluency were transfected with 5.0 μg HNF4a plasmid (Addgene, #31100) and 5.0 μg pCruz-HA-AMOT plasmid (WT or p.S50G) per dish for 24 h. Co-immunoprecipitation was performed using the Immunoprecipitation Kit (Roche, Germany) according to the manufacturer’s instructions. In brief, the cotransfected HEK293 cells were lysed in 1 ml of cold Lysis Buffer containing Protease Inhibitor Cocktail for 30 min. After removal the cell debris by centrifugation and preclearing with 50 μl of the Protein G Sepharose beads, whole cell lysate was incubated with 2μg of antibody and protein G Sepharose beads overnight at 4 ℃. The immunocomplexes were then washed with lysis buffer and IP washing buffers sequentially and separated by SDS-PAGE. Immunoblotting was performed following standard procedures.

## ***Western blot***

Protein samples were harvested in ice-cold RIPA Buffer (ThermoScientific) containing freshly added protease inhibitors. After brief sonication on ice, cell lysates were centrifuged at 14,000 *g* for 15 min at 4 ℃ to remove cell debris. The supernatant was collected and protein content was quantified using a Pierce® BCA Protien Assay Kit (ThermoScientific) according to the manufacturer’s instruction. Protein mixture in cell lysates was separated using SDS-PAGE and transferred to a nitrocellulose membrane (Bio-Rad Laboratories). The transblotted membrane was blocked with 5% (w/v) non-fat milk, incubated with the primary antibodies, and appropriate secondary antibodies. The antibody/protein complexes were visualized using the enhanced chemiluminescence system (Perkin-Elmer).

***Metabolomic profiling of rat plasma by GC-MS/MS analysis***

A total 30 μl of plasma was collected from 1-month old rats, and 20 μl of myristic-d_27_ acid (200 μg/mL, dissolved in methanol; Sigma-Aldrich) and 200 μl of methanol were added. After the sample was vortexed for 5 min and centrifuged for 10 min at 15,000 *g*, 200 μl of supernatant was transferred to a glass centrifuge tube and dried under nitrogen gas. The dried samples were re-suspended in 100 μl of toluene, vortexed vigorously for 10 s, and dried again under nitrogen. A two-step derivatization method was used for chemical derivatization of the metabolites. Samples were first incubated with 50 μl of 2% methoxy-amine chloride (Thermo Fisher Scientific) in pyridine for 1.5 h at 60°C. Next, 50 μl of *N*-methyl-*N*-trifluoroacetamide (Thermo Fisher Scientific) was added and the samples were incubated for 1 h at 60°C. A total of 80 μl of derivatized samples were transferred to glass vials for GC-MS/MS analysis. Pooled rat plasma from each sample were used as QC and distilled water as blank control. GC-MS/MS analysis was performed using a Shimadzu TQ8040 gas chromatography-triple quadrupole mass spectrometer (Shimadzu Corporation, Japan). The GC injector port was set at 250°C and injection volume was 1 μl with a spilt ratio of 1:10. Chromatographic separation was achieved by a capillary column (BPX-5, 30 m × 0.25 mm × 0.25 μm; SGE Analytical Science, Australia) through constant helium carrier gas flow at 1.14 mL/min. The oven temperature was first held at 60°C for 2 min, followed by increasing temperature ramp of 15°C/min to 330°C, and then held at 330°C for 3 min. The interface and ion source were set at 280 and 200°C, respectively. The mass spectrometer was operated in the multiple reaction monitoring (MRM) mode.^15^

***Metabolite identification and data processing***

The Shimadzu Smart Metabolites Database (Shimadzu Corporation, Japan) which contains a panel of 475 different endogenous metabolites was applied to identify the metabolites in rat plasma. This database, which facilitates rapid and reliable metabolite identification and semi-quantification, contains the retention indices and two MRM transitions of each of the 475 endogenous metabolites.^16-18^ Metabolites in samples with peak areas at least fivefold of those in blank samples were selected for data analysis. In addition, metabolites with ion transition ratio variation > 30% and/or with peak area variation in QC samples > 30% were considered unstable and removed from analysis.^19^ The raw data were normalized with the peak area of internal standard (myristic-d_27_ acid) and the final data were imported into the SIMCA 13® software (Umetrics AB; Sweden). Metabolites were subjected to *t*-test with a *P*-value of less than 0.05 indicating statistical significance. Furthermore, FDRs were calculated by Benjamini-Hochberg procedure. Metabolites with FDR < 0.1 and FC > 1.15 or < 0.85 were considered as significant.

***Statistics***

Unless annotated otherwise, each experiment was carried out at least three times, with similar outcomes. Statistical analyses were performed using SPSS (version 25.0), and comparisons between the groups were performed using Mann-Whitney *U* test. A *P* value of less than 0.05 was considered significant.

***Study approval***

Animal experiments were conducted under protocols approved by the Institutional Animal Care and Use Committee (IACUC) of National University of Singapore (Protocol No. BR24/12, BR13-6278 and R13-6265).

**Tables**

## **Table S1. Antibodies used in this study.**

| **Antibody** | **Host** | **Supplier** | **Cat. No** |
| --- | --- | --- | --- |
| **Immunohistochemistry** | | | |
| HNF4α | Rabbit | Thermo Fisher Scientific | PA5-79380 |
| **Immunofluorescence** | | | |
| ZO-1 | Rabbit | Thermo Fisher Scientific | 40-2200 |
| HNF4α | Rabbit | Thermo Fisher Scientific | PA5-79380 |
| Occludin | Rabbit | Thermo Fisher Scientific | 40-4700 |
| E-cadherin | Rabbit | ProteinTech Group | 20648-1-AP |
| Rabbit IgG (H&L)-FITC | Goat | Abcam | ab6717 |
| Rabbit IgG (H&L)-PE | Goat | Thermo Fisher Scientific | A11011 |
| Phalloidin-Alexa Fluor 488 | | Thermo Fisher Scientific | A12379 |
| **Co-immunoprecipitation/Western blot** | | | |
| Amot | Rabbit | Thermo Fisher Scientific | PA5-31196 |
|  | Mouse | Santa Cruz Biotechnology | sc-166924 |
| Beta-actin | Mouse | ProteinTech Group | 66009-1-IG |
| HA | Rabbit | Santa Cruz | sc-805 |
| HNF4a | Rabbit | Thermo Fisher Scientific | PA5-79380 |
| Mouse IgG-HRP | Rabbit | Santa Cruz Biotechnology | sc-358917 |
| Myc | Mouse | Thermo Fisher Scientific | MA1-980 |
| Rat IgG (H+L)-HRP | Goat | ProteinTech Group | SA00001-15 |
| **Urine albumin Elisa** | | | |
| Anti-rat albumin antiserum | Rabbit | MP Biomedicals | 0955711 |
| Anti-rat albumin IgG fraction-HRP | Sheep | MP Biomedicals | 0855776 |
| **Albumin flux** | | | |
| Albumin, FITC conjugate | Bovine | Thermo Fisher Scientific | A23015 |

## **Table S2. Primers sequence.**

| **Gene** | **Primers** | | **Product length** |
| --- | --- | --- | --- |
| *AMOT*_human  NM_001113490 | F: | AACCTCGTGAAGTCATCCTC | 239 bp |
|  | R: | TCCGCTTCCAGCTTCTCCTT |  |
| *GAPDH*_human  NM_002046 | F: | CTGGCATGGCCTTCCGTGTC | 194 bp |
|  | R: | GGAGGAGTGGGTGTCGCTGT |  |
| *Amo*t_rat  XM_006257420 | F: | CCAGCAAGCCACAGGGAATA | 185 bp |
|  | R: | TTCGAGGGGACACTTGCTTC |  |
| *Amot*_rat gDNA  NC_051356 | F: | CTGTTTCCCCTTCCCAGGTC | 518 bp |
|  | R: | GACGTCCCTCGGTTCTCATC |  |
| *Acox2*_rat  NM_145770 | F: | CTTCATGACCCGAGAGGAGC | 183 bp |
|  | R: | CCCAGGCTTCGGATAGCATT |  |
| *Aldh2*_rat  NM_032416 | F: | TCCGCTATTATGCTGGCTGG | 180 bp |
|  | R: | CCACGTTTCCAGTTGCCAAG |  |
| *Aldob*_rat  NM_012496 | F: | CCTTCCAGCCTTGCTATCCAA | 209 bp |
|  | R: | AGGGTGCCCTCAAGGTAAAC |  |
| *Oct*_rat  NM_013078 | F: | AGACATTCACTTGGGCGTGA | 211 p |
|  | R: | GCCATAGTGTTCCTGGAGTGT |  |
| *Gadph*_rat  NM_017008 | F: | CGTGTTCCTACCCCCAATGT | 214 bp |
|  | R: | CATTGAGAGCAATGCCAGCC |  |

## **Table S3. List of Hnf4α target genes in rat*.**

| *Acaa2* |
| --- |
| *Acadm* |
| *Acox1* |
| *Acox2* |
| *Acox3* |
| *Acoxl* |
| *Agt* |
| *Aldh2* |
| *Aldh3a1* |
| *Aldh3a2* |
| *Aldh3b1* |
| *Aldob* |
| *Ambp* |
| *Apoa1* |
| *Apoa2* |
| *Apoa4* |
| *ApoB* |
| *Apoc2* |
| *Apoc3* |
| *Apoe* |
| *Ceacam1* |
| *Cfb* |
| *Cyp3a1* |
| *Cyp3a23* |
| *Cyp7a1* |
| *Dhdh* |
| *Epo* |
| *F10* |
| *F7* |
| *F8* |
| *F9* |
| *Fabp2* |
| *Ghr* |
| *Gucy2c* |
| *Hadh* |
| *Hmgcr* |
| *Hnf1a* |
| *Mst1* |
| *Mttp* |
| *Onecut1* |
| *Otc* |
| *Pck1* |
| *Pck2* |
| *Pklr* |
| *Prlr* |
| *Rbp2* |
| *Serpina1* |
| *Shbg* |
| *Tat* |
| *Tf* |
| *Ttr* |

*Adapted from Dr. Frances M. Sladek' reports. https://sladeklab.ucr.edu/hnf43.pdf

## **Table S4.**

**Critical metabolites in rat plasma associated with *Amot* mutation.** Metabolic profiling of the plasma of one-month-old rats (n = 10 for each group) using GC-MS/MS metabolites analysis platform. A total 116 metabolites passed the QC criteria. Based on the well accepted standard of FDR < 0.1 and FC > 1.15 or < 0.85, thirty-seven metabolites were identified as critical metabolites as a result of the PG mutation. Among the 37 critical metabolites, 33 metabolites were up-regulated and 4 metabolites were down-regulated.

| No | Metabolite | *P*-value | FDR | Log2 (Fold Change) |
| --- | --- | --- | --- | --- |
| 1 | 2-Hydroxyisobutyric acid | < 0.001 | < 0.001 | 2.003 |
| 2 | Fructose | < 0.001 | < 0.001 | 1.006 |
| 3 | Norepinephrine | < 0.001 | < 0.001 | 0.566 |
| 4 | Psicose | < 0.001 | < 0.001 | 1.014 |
| 5 | Sorbose | < 0.001 | < 0.001 | 1.005 |
| 6 | Tagatose | < 0.001 | < 0.001 | 0.997 |
| 7 | Isocitric acid | 0.001 | 0.006 | 0.939 |
| 8 | 4-Hydroxybenzoic acid | 0.001 | 0.006 | 0.903 |
| 9 | Acetylglycine | 0.001 | 0.007 | 0.739 |
| 10 | Xylose | 0.001 | 0.008 | 0.588 |
| 11 | 2-Aminopimelic acid | 0.002 | 0.008 | 1.113 |
| 12 | Citric acid | 0.002 | 0.009 | 1.11 |
| 13 | Arabinose | 0.002 | 0.010 | 0.884 |
| 14 | Lyxose | 0.002 | 0.010 | 0.555 |
| 15 | Octanoic acid | 0.002 | 0.011 | -0.538 |
| 16 | Isoleucine | 0.003 | 0.012 | 0.819 |
| 17 | Glutaric acid | 0.004 | 0.014 | 0.951 |
| 18 | Succinic acid | 0.004 | 0.015 | 0.663 |
| 19 | Decanoic acid | 0.005 | 0.017 | -0.456 |
| 20 | Lactitol | 0.005 | 0.017 | -0.871 |
| 21 | Uracil | 0.009 | 0.027 | 0.456 |
| 22 | Allose | 0.011 | 0.032 | 0.255 |
| 23 | 3-Phenyllactic acid | 0.019 | 0.052 | 1.056 |
| 24 | 2-Hydroxyglutaric acid | 0.019 | 0.053 | 0.532 |
| 25 | Creatinine | 0.023 | 0.056 | 0.939 |
| 26 | Ribonic acid | 0.022 | 0.058 | 1.144 |
| 27 | Indol-3-acetic acid | 0.023 | 0.058 | 0.659 |
| 28 | 3-Hydroxybutyric acid | 0.029 | 0.065 | 0.563 |
| 29 | Xylulose | 0.029 | 0.067 | 0.453 |
| 30 | Homoserine | 0.031 | 0.067 | 0.426 |
| 31 | Lauric acid | 0.029 | 0.069 | -0.418 |
| 32 | Glycerol 3-phosphate | 0.035 | 0.070 | 0.531 |
| 33 | Ribulose | 0.035 | 0.072 | 0.512 |
| 34 | Malic acid | 0.035 | 0.074 | 0.501 |
| 35 | Alanine | 0.043 | 0.078 | 0.587 |
| 36 | Quinolinic acid | 0.043 | 0.080 | 0.533 |
| 37 | 3-Hydroxyisovaleric acid | 0.043 | 0.082 | 0.228 |

**Figures**

## **
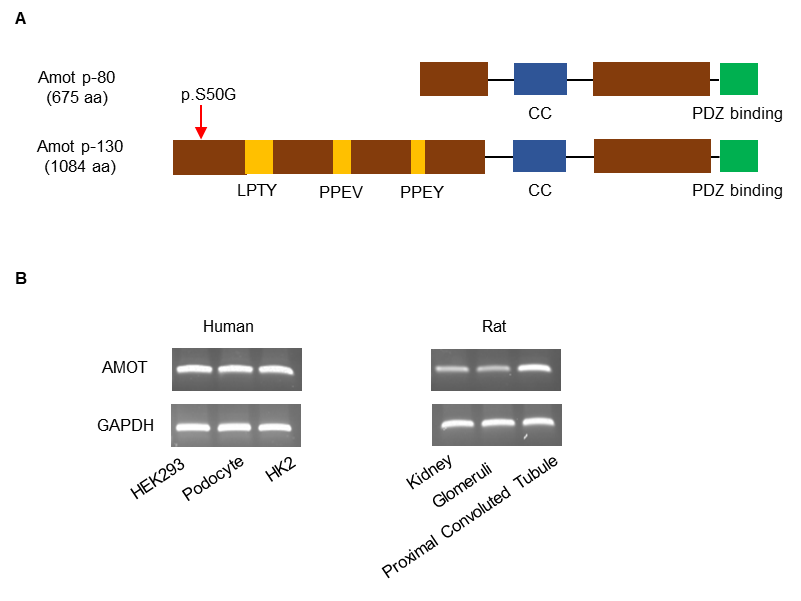
Figure S1**

**Figure S1. Structure of angiomotin proteins and expression of AMOT-P130 in kidneys. (A)** Angiomotin (*AMOT*) encodes two isoforms, AMOT-P80 and -P130, by alternative splicing. While P130 have a N-terminus extension, both of the isoforms share identical C-terminus that contain conservative coiled-coil (CC) domains and C-terminal PDZ domain. In addition, three L/P-PXY motifs (106-109 aa in LPTY; 239-242 aa in PPEY, and 284-287 aa in PPEY) are located in the N-terminus of AMOT-p130. The mutation p.S50G located at the N-terminus of AMOT-p130 lies upstream of all reported motifs. **(B)** Reverse transcription PCR on human kidney cell lines (namely human embryonic kidney (HEK)-293, podocyte and human tubular cells (HK2)), as well as rat kidneys (namely whole kidney, glomeruli and proximal convoluted tubules) confirmed AMOT expression in kidney.

## **
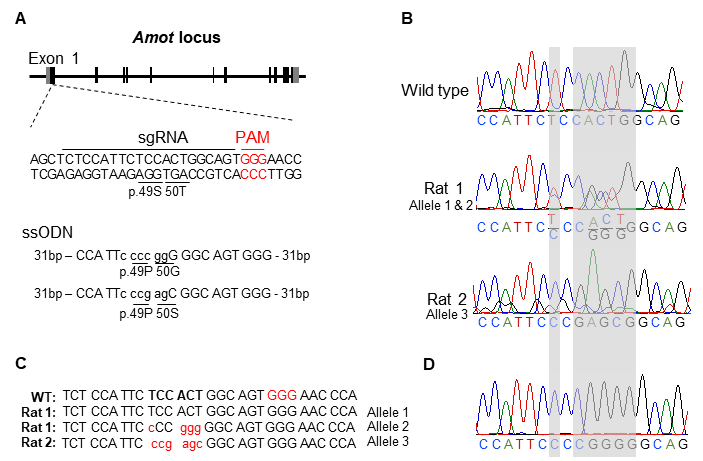
Figure S2**

**Figure S2.** **Establishment of *Amot* mutant rat lines.** **(A)** Schematic diagram of the rat *Amot* gene locus with 11 exons. The target sequence in exon 1 chosen to generate the single guide RNA (sgRNA) is shown. Protospacer adjacent motif (PAM) is indicated in red. PG genetic variant refers to substitution of serine (S) and threonine (T) at positions 49-50 with proline (P) and glycine (G) respectively. PS genetic variant refers to substitution of serine (S) and threonine (T) at positions 49-50 with proline (P) and serine (S) respectively. Two single-stranded donor oligonucleotides (ssODN), which encoded the PG and PS *Amot* genetic variants served as templates for homologous recombination mediated repair. An AvaI restriction cut site, indicated in lower case, was introduced to aid genotyping. Grey boxes represent untranslated regions while black boxes represent protein-coding regions. **(B)** Two founder rats were genotyped *via* sanger sequencing. Rat 1 (female) was confirmed to be heterozygous for PG variant while Rat 2 (female) was confirmed homozygous for PS variant. **(C)** Allele sequences for the two founder rats. **(D)** Sanger sequencing on hemizygous PG rats.

## **Figure S3**


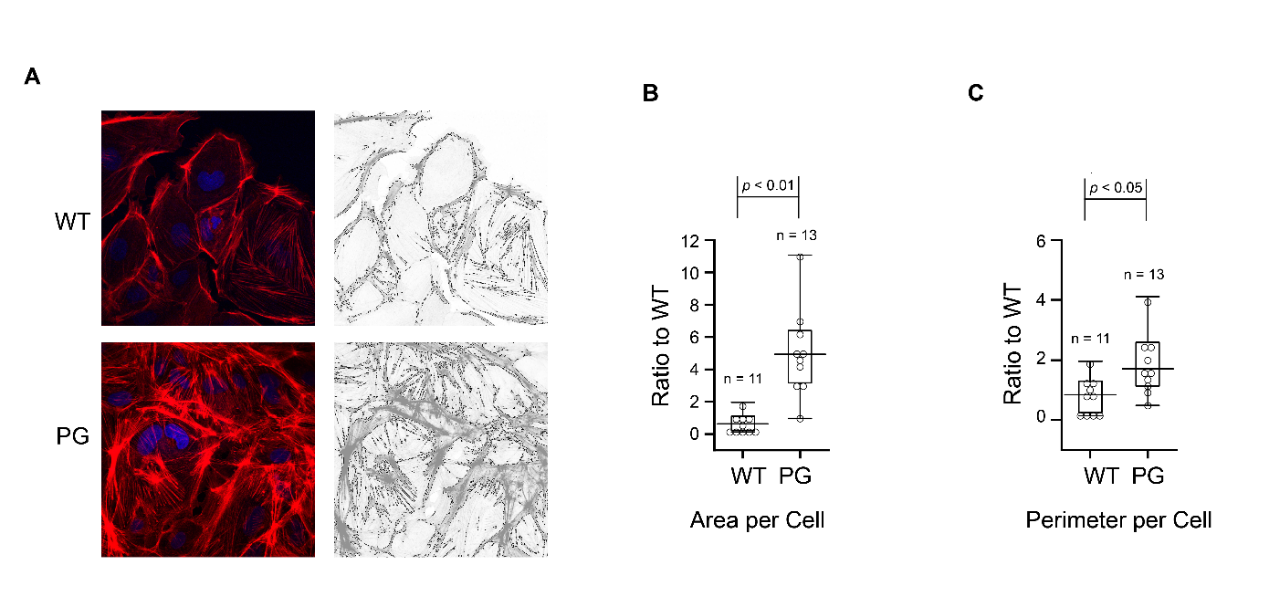


**Figure S3. Immunofluorescence of *ex vivo* podocytes by phalloidin staining. (A)** Representative F-actin stain showed the PG mutation caused abnormal stress fiber formation. ImageJ was used to quantify the stress fibre area. **(B)** Results from 11 WT images and 13 PG images showed increased stress fibre areas (P < 0.01) in PG podocytes compared to WT podocytes.

## **Figure S4**


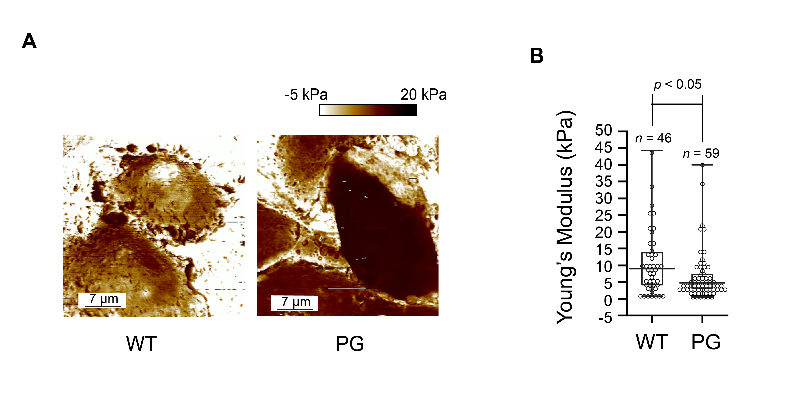


**Figure S4. Amot PG genetic alteration reduced cell stiffness on *ex vivo* podocytes.** Cell stiffness were measured with atomic force microscope (AFM). The reduction of cell stiffness was qualitatively demonstrated by the colour transition from bright to dark in Derjaguin-Muller-Toporov (DMT) Modulus **(A)**. This was quantitatively compared using Young’s Modulus **(B)**.

## **Figure S5.**

**
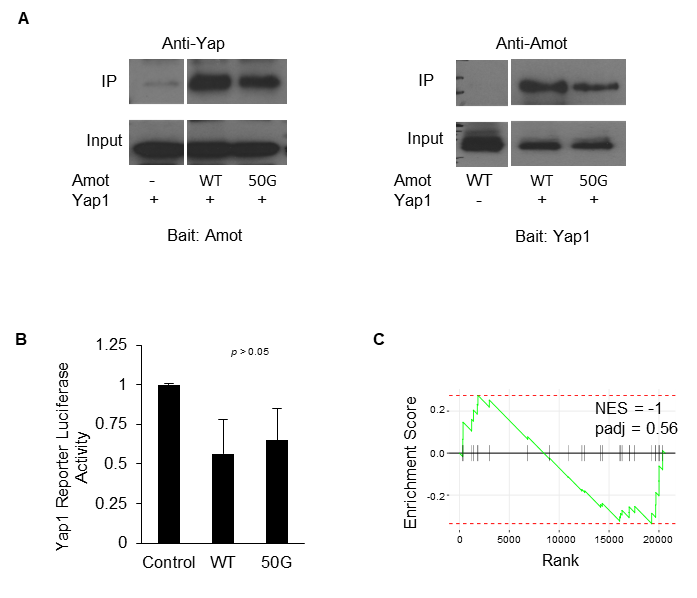
Figure S5. Hippo signalling pathway was not affected by the *AMOT* mutation. (A)** Co-immunoprecipitation of AMOT-130 with YAP. Co-IP results showed YAP was pulled down by AMOT (left), *vice versa*, AMOT was pulled down by YAP (right). No difference was observed in the affinity with YAP between WT and p.S50G AMOT. **(B)** In the dual-luciferase reporter system, the inhibition effects of AMOT on YAP/TAZ-responsive promoter driving luciferase expression were not reduced by the p.S50G genetic alternation. **(C)** Gene Set Enrichment Analysis (GSEA) with a set of evolutionary conserved YAP target genes (MSigDB; M2871) showed the YAP target genes were not up-regulated in the PG rats (adjusted *P* > 0.05).

## **Figure S6.**

**
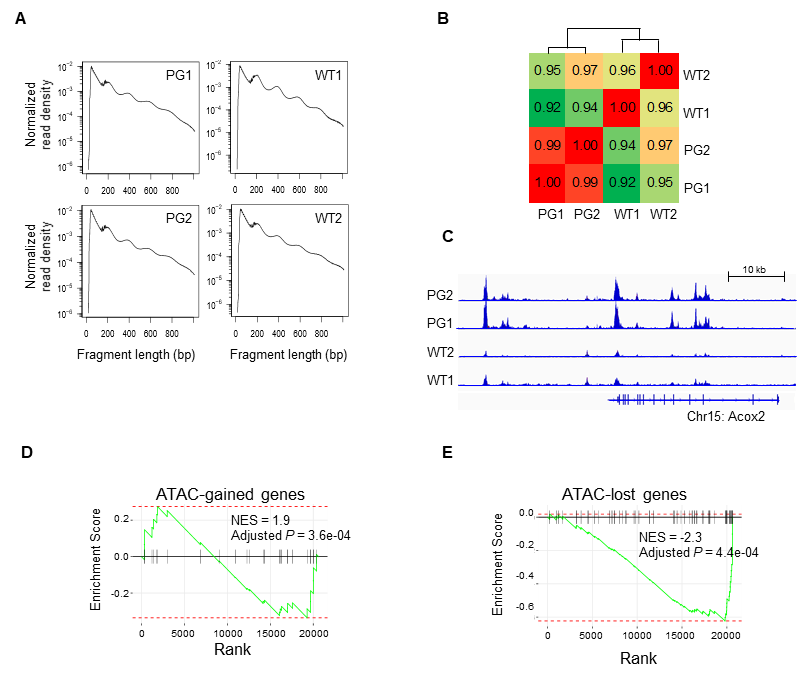
Figure S6. ATAC-seq on freshly isolated proximal convoluted tubular cells from rats.** **(A)** The ATAC-seq signal enrichment around the transcription start sites showed the typical characteristics of successful ATAC-seq for all four samples (two PG and two WT rats). **(B)** Heatmap of Pearson correlation between the samples. Hierarchical clustering was used to group similar samples into two clusters. Correlation coefficients between each two samples were shown in the boxes crossed by the two samples. Correlation coefficient values were reflected by the box colours (the lowest to the highest in value, green to red in colour). **(C)** Representative sequencing tracks for the *Acox2* locus showed distinct ATAC-seq peaks at the promoter and the enhancer regions. The ATAC-seq data had been normalized to take sequencing depth into account and the scale on the y-axis was chosen for optimal visualization of peaks for each sample. **(D)** GSEA analysis on RNA-Seq results showed ATAC-gained region genes were upregulated (NES =1.9, adjusted *P* = 0.00036) **(E)** while the ATAC-lost region genes were down-regulated (NES = -2.3, adjusted *P* = 0.00044) in PG mutate rats.

## **
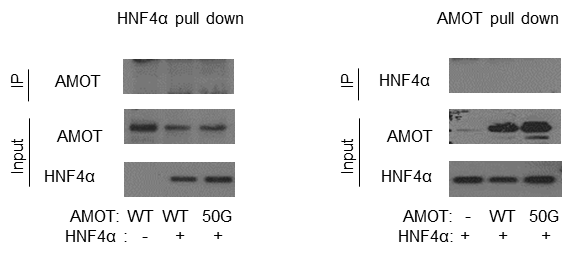
Figure S7.**

**Figure S7. Co-immunoprecipitation of AMOT-130 with HNF4α.** Co-IP results showed neither the wild type nor the mutant AMOT-P130 was not pulled down by HNF4α (left), *vice versa*, HNF4α was not pulled down by neither the wild type nor the mutant AMOT-P130 (right).

## **Figure S8.**


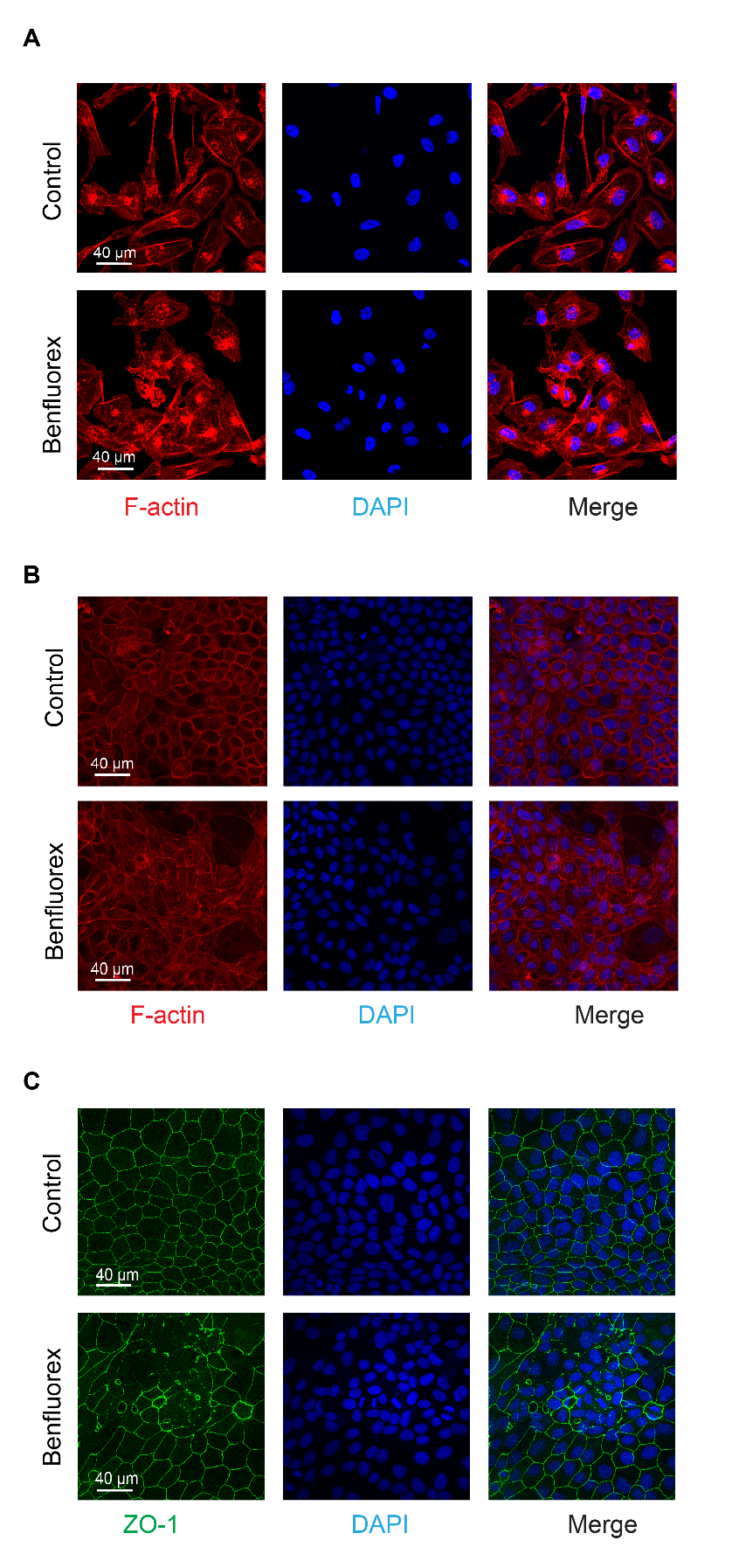


**Figure S8. Hnf4α activation caused abnormal stress fiber formation and tight junction disturbance in wild-type kidney cell lines.** Immunofluorescence of F-actin in human tubular cells HK-2 (**A**) and Madin-Darby canine kidney (MDCK) cells (**B**) treated with Benfluorex, a known Hnf4α activator suggested that Hnf4α activation induced stress fibre formation with associated cytoskeletal disorganisation. The distribution of the tight junction protein ZO-1 was also disturbed in MDCK cells treated with Benfluorex (**C**).

## **Figure S9.**

**
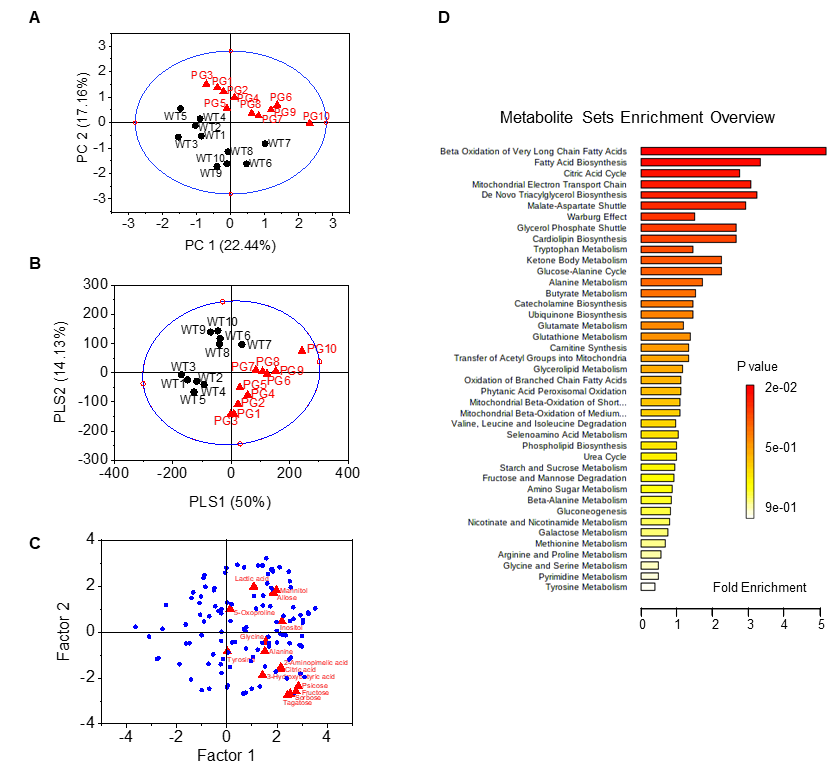
Figure S9. Principal component and partial least squares discriminant analysis of metabolic profile in rat plasma.** **(A)** Principal component analysis (PCA) of the identified metabolites in rat plasma. PC1 is the first principal component that distinguished the WT (black) and PG (red) groups while PC2 is the second principal component. PC1 could explained 22.44 % of the variation while PC2 explained 17.16 % of the variation. **(B)** Partial least squares discriminant analysis (PLS-DA) of metabolite profile in the plasma of WT (black) and PG (red) rats. PLS1 is the first principal component that distinguished WT and PG groups while PLS2 is the second principal component. **(C)** Loading plot of the 131 commonly detected compounds projected into the PLS-DA model. The most important compounds (Variable Importance in Projection (VIP) > 1.2) responsible for the discrimination are shown as red triangles, and the other compounds are shown as blue dots. **(D)** Pathway enrichment of critical metabolites with MetaboAnalyst®. Plots depicting computed metabolic pathways as a function of –log (*P*) are shown.

**Reference**

1. Shao Y, Guan Y, Wang L, et al. CRISPR/Cas-mediated genome editing in the rat via direct injection of one-cell embryos. *Nat Protoc*. 2014;9(10):2493-2512.

2. Rush BM, Small SA, Stolz DB, Tan RJ. An Efficient Sieving Method to Isolate Intact Glomeruli from Adult Rat Kidney. *J Vis Exp*. 2018;(141):10.3791/58162.

3. Baer PC, Nockher WA, Haase W, Scherberich JE. Isolation of proximal and distal tubule cells from human kidney by immunomagnetic separation. Technical note. *Kidney Int*. 1997;52(5):1321-1331.

4. Ding W, Yousefi K, Shehadeh LA. Isolation, Characterization, And High Throughput Extracellular Flux Analysis of Mouse Primary Renal Tubular Epithelial Cells. *J Vis Exp*. 2018;(136):57718.

5. Cooper JA, Del Vecchio PJ, Minnear FL, et al. Measurement of albumin permeability across endothelial monolayers in vitro. *J Appl Physiol (1985)*. 1987;62(3):1076-1083.

6. Li B, Dewey CN. RSEM: accurate transcript quantification from RNA-Seq data with or without a reference genome. *BMC Bioinformatics*. 2011;12:323.

7. Love MI, Huber W, Anders S. Moderated estimation of fold change and dispersion for RNA-seq data with DESeq2. *Genome Biol*. 2014;15(12):550.

8. Ashburner M, Ball CA, Blake JA, et al. Gene ontology: tool for the unification of biology. The Gene Ontology Consortium. *Nat Genet*. 2000;25(1):25-29.

9. Kanehisa M, Goto S. KEGG: kyoto encyclopedia of genes and genomes. *Nucleic Acids Res*. 2000;28(1):27-30.

10. Yu G, Wang LG, Han Y, He QY. clusterProfiler: an R package for comparing biological themes among gene clusters. *OMICS*. 2012;16(5):284-287.

11. Buenrostro JD, Wu B, Chang HY, Greenleaf WJ. ATAC-seq: A Method for Assaying Chromatin Accessibility Genome-Wide. *Curr Protoc Mol Biol*. 2015;109:21 29 21-21 29 29.

12. Langmead B, Salzberg SL. Fast gapped-read alignment with Bowtie 2. *Nat Methods*. 2012;9(4):357-359.

13. Shabana, Shahid SU, Hasnain S. Use of a gene score of multiple low-modest effect size variants can predict the risk of obesity better than the individual SNPs. *Lipids Health Dis*. 2018;17(1):155.

14. Ramirez F, Dundar F, Diehl S, Gruning BA, Manke T. deepTools: a flexible platform for exploring deep-sequencing data. *Nucleic Acids Res*. 2014;42(Web Server issue):W187-191.

15. Dai Y, Yeo SCM, Barnes PJ, et al. Pre-clinical Pharmacokinetic and Metabolomic Analyses of Isorhapontigenin, a Dietary Resveratrol Derivative. *Front Pharmacol*. 2018;9:753.

16. Hashimoto K, Ishima T, Sato Y, et al. Increased levels of ascorbic acid in the cerebrospinal fluid of cognitively intact elderly patients with major depression: a preliminary study. *Sci Rep*. 2017;7(1):3485.

17. Nishiumi S, Kobayashi T, Kawana S, et al. Investigations in the possibility of early detection of colorectal cancer by gas chromatography/triple-quadrupole mass spectrometry. *Oncotarget*. 2017;8(10):17115-17126.

18. Tomonaga S, Okuyama H, Tachibana T, Makino R. Effects of high ambient temperature on plasma metabolomic profiles in chicks. *Anim Sci J*. 2018;89(2):448-455.

19. Chen L, Cheng CY, Choi H, et al. Plasma Metabonomic Profiling of Diabetic Retinopathy. *Diabetes*. 2016;65(4):1099-1108.
